# Early changes in renal resistive index and mortality in diabetic and nondiabetic kidney transplant recipients: a cohort study

**DOI:** 10.1186/s12882-021-02263-8

**Published:** 2021-02-19

**Authors:** Jean-Baptiste de Freminville, Louis-Marie Vernier, Jérome Roumy, Frédéric Patat, Philippe Gatault, Bénédicte Sautenet, Christelle Barbet, Hélène Longuet, Elodie Merieau, Matthias Buchler, Jean-Michel Halimi

**Affiliations:** 1 Néphrologie-Immunologie Clinique, Hôpital Bretonneau, CHU Tours, Tours, France; 2 University of Tours, Tours, France; 3 Néphrologie-Dialyse, Centre de santé pluridisciplinaire, Le Mans, France; 4 Imagerie Médicale, Hôpital Bretonneau, CHU Tours, Tours, France; 5 CIC-IT 1415, CHU Tours, Tours, France; 6 EA4245, University of Tours, Tours, France

**Keywords:** Renal resistive index, Diabetes mellitus, Ultrasonography, Kidney transplantation

## Abstract

**Background:**

Renal resistive index (RI) predicts mortality in renal transplant recipients (RTR). However, its predictive value may be different according to the time of measurement. We analysed RI changes between 1 month and 3 months after transplantation and its predictive value for death with a functioning graft (DWFG).

**Methods:**

We conducted a retrospective study in 1685 RTR between 1985 and 2017. The long-term predictive value of changes in RI value from 1 month to 3 months was assessed in diabetic and non-diabetic RTR.

**Results:**

Best survival was observed in RTR with RI < 0.70 both at 1 and 3 months, and the worst survival was found in RTR with RI ≥ 0.70 both at 1 and 3 months (HR = 3.77, [2.71–5.24], *p* < 0.001). The risk of DWFG was intermediate when RI was < 0.70 at 1 month and ≥ 0.70 at 3 months (HR = 2.15 [1.29–3.60], *p* = 0.003) and when RI was ≥0.70 at 1 month and < 0.70 at 3 months (HR = 1.90 [1.20–3.03], *p* = 0.006).

In diabetic RTR, RI was significantly associated with an increased risk of death only in those with RI < 0.70 at 1 month and ≥ 0.70 at 3 months (HR = 4.69 [1.07–20.52], *p* = 0.040). RI considered as a continuous variable at 1 and 3 months was significantly associated with the risk of DWFG in nondiabetic but not in diabetic RTR.

**Conclusion:**

RI changes overtime and this impacts differently diabetic and nondiabetic RTR. RI short-term changes have a strong prognosis value and refines the risk of DWFG associated with RI.

**Supplementary Information:**

The online version contains supplementary material available at 10.1186/s12882-021-02263-8.

## Background

Kidney transplantation is unquestionably the best treatment of end-stage renal disease (ESRD), but kidney transplant recipients have a higher mortality rate than the general population [1]. In a seminal study, Radermacher et al. found that high renal Resistive Index (RI) measured at least 3 months after renal transplantation was associated with an increased risk of death [2]. However, the timing of RI measurements in this study was very variable, with a median of 40 months. Naesens et al. confirmed its predictive value on the risk of death in renal transplantation at different time-points (3, 12 and 24 months [3].

However, some caution may be applied regarding the use of RI to assess the risk of death. First, we reported that high RI at 3 months was not associated with an increased risk of death in a large cohort of diabetic renal transplant recipients (RTR), so the prognostic value of RI may be different in diabetic as compared to nondiabetic RTR [4]. Second, the timing of RI measurement may impact its prognostic value: it was demonstrated that RI measured on the early post-transplant period (between the second and fourth day after transplantation) [5], and RI measured before 12 months after transplantation, were not associated with the risk of death in some studies [6]. These findings may suggest that the RI value can change overtime in some patients, and that one single measurement may be insufficient to accurately evaluate the risk of death.

In the present study, we analysed RI changes between 1 month and 3 months after transplantation, and we assessed its long-term predictive value for death with a functioning graft (DWFG) in a large cohort of diabetic and non-diabetic renal transplant recipients.

## Materials and methods

### Patient selection

**Fig. 1:**
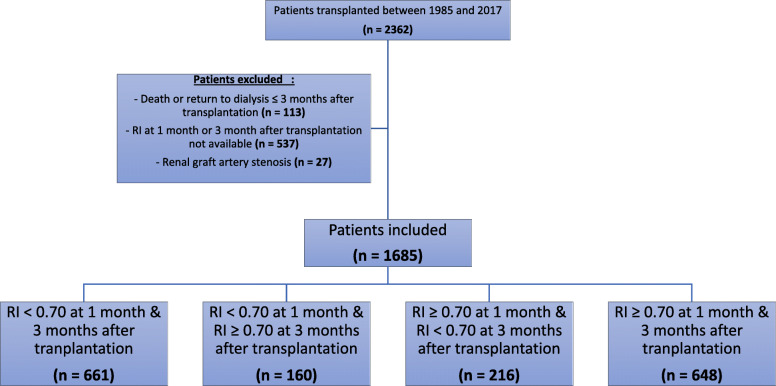
Flow chart diagram

We conducted a retrospective analysis of 2362 consecutive patients who received a renal transplant from October 1985 to October 2017 at the Tours University Hospital, France. Among them, 113 died or returned to dialysis within the three first months following transplantation, 537 patients were excluded because renal Doppler ultrasonography at 1 month or at 3 months after transplantation was not available, and 27 were excluded because of a diagnosis of renal graft artery stenosis (Fig. 1). Thus, 1685 patients were included in this study. Data were collected from our prospectively maintained institutional database of transplant patients and the ASTRE database [“commission nationale informatique et liberté” (CNIL) agreement number: DR-2012-518]. The study protocol was validated by the Ethics Committee in Human Research (Hôpital Bretonneau, CHU Tours, France) and is in accordance with the Helsinki declaration of 1975, as revised in 2013. Results are reported according to the STROBE Statement [7].

Visits in our hospital for the follow-up were organized as follows: three visits per week during the first 2 weeks; two visits per week during the first month; one visit per week during the three first months; one visit per month during the first year; one visit every other month during the second year; three visits per year thereafter until death, or ESRD (i.e., dialysis or re-transplantation).

At the time of transplantation, the following variables were reviewed: donor age, gender, diabetes, double or single transplantation, machine perfusion; recipient age, gender, diabetes, graft rank, body mass index (BMI), hemodialysis time before transplantation. At the 3-month visit after transplantation, the following variables were reviewed: systolic, diastolic and pulse arterial pressure, serum creatinine level, estimated glomerular filtration rate (eGFR) (using MDRD equation), proteinuria (by a 24-h urine collection [8]) immunosuppressive induction and maintenance treatments, delayed graft function (DGF) after transplantation, and RI. Regarding immunosuppressive induction treatment, patients received T-cell depletion therapy or basiliximab according to the protocol of our service (systematic T-cell therapy in case of Donor Specific Antigen (DSA), donor cardiac arrest type Maastricht 2, according to immunological risk in other situations). For double transplantation, RI was the mean of both left and right graft RI value. Recipient diabetes was defined as diabetes diagnosed before transplantation; it did not include new-onset diabetes after transplantation (NODAT). NODAT was defined according to the American Diabetes Association (ADA): symptoms of diabetes plus casual plasma glucose concentration > 11.1 mmol/L, casual being defined as any time of day without regard to time since last meal or fasting glucose > 7 mmol/l, fasting being defined as no caloric intake for at least 8 h (oral glucose tolerance tests were not usually performed in our centre, because they are not recommended in routine practice). These criteria were confirmed by repeat testing on a different day. Cardiovascular death for the donor was defined as death from cardiac or cerebrovascular cause.

### Doppler ultrasonography studies

Renal RI is studied in our hospital since the early seventies [9]. For the measurement, three ultrasound systems were used: Toshiba Aplio XG with PVT-375BT probe, Esaote Technos MPX with probe and Siemens Antares Premium Edition with CH5–2 probe with vascular programme for each exam [10]. Peak systolic velocity (PSV) and end-diastolic velocity (EDV) were measured during Doppler ultrasonography spectral analysis in renal interlobar arteries at three different points of the kidney (upper, medium and lower). RI was calculated with PSV and EDV by the following equation:

$$ RI=\raisebox{1ex}{$\left( PSV- EDV\right)$}\!\left/ \!\raisebox{-1ex}{$ PSV$}\right. $$

The mean of three consecutive measurements was used. Doppler ultrasonography studies were routinely performed at 1 month and 3 months after transplantation. Renal artery stenosis was ruled out at the time of measurements. The results of other Doppler studies were not considered in this report.

### Statistical analyses

All the variables had a normal distribution. Results are expressed as percentages or means ± standard deviations. Qualitative variables were compared using Chi-square test. Continuous variables were compared between two groups using Student t test and between multiple groups using analysis of variance (ANOVA).

The patients were stratified in four groups depending on the value of RI at 1 month and at 3 months after transplantation. We used 0.70 as cut-off because it was the closest value from the mean and the median of RI in our cohort. Moreover, some studies consider 0.70 as the upper threshold of normal RI [11, 12], whereas others showed that a RI greater than 0.75 or 0.80 was associated with death [2, 13, 14]. We used 0.75 as cut-off in sensitivity analyses. We did not use 0.80 as a cut-off because too few patients had a RI of more than 0.80.

To assess colinearity among the variables, we used Pearson correlation.

For survival analysis, the event of interest was death with a functioning graft (DWFG). As graft loss (i.e. dialysis or re-transplantation) are events that hinder the observation of the event of interest, and are competing risks, we used the cumulative incidence competing risk (CICR) method. To assess the association between RI at 1 month and 3 months and the risk of DWFG, we compared cumulative incidence functions, using the subdistribution hazard approach proposed by Fine and Gray [15] in univariate and multivariate analysis, after analyzing the effect of multiple variables on the risk of DWFG, in order to choose the confounding factors. Variables associated with DWFG in univariate were identified as potential confounders and included in multivariate analysis. We also assessed RI at 1 month and RI at 3 months after transplantation as continuous variables in splines-based hazard ratio curves [16].

A *p* value < 0.05 was considered statistically significant. Analyses were performed using the statistical software RStudio (RStudio Team, 2015, v1.0.153).

## Results

### Baseline characteristics

Median follow-up was 6.36 years (0.25 to 30.9 years; total observation period: 13,427 patient years).

Among these 1685 renal transplant recipients, 821 patients (48.7%) at 1 month, and 877 patients (52.0%) at 3 months had a RI of less than 0.70 (Table 1). 263 patients had pre-transplant diabetes. It was the first transplantation for 1433 patients (85.0%). 1590 patients (94.4%) received a cadaveric graft and 924 (61.4%) received a kidney from a donor deceased from cardiovascular disease (Table 1). Regarding immunosuppression, patients first received anti-interleukin 2 receptor (44.3%) or thymoglobulin (54.4%), and methylprednisolone 250 mg before and after transplantation. Their treatment then included prednisone and mycophenolate mofetyl (81.0%) or azathioprine (15.9%), associated with ciclosporin (39.8%), tacrolimus (55.9%) or mechanistic target of rapamycin (m-TOR) inhibitors (6.6%) (Table 1).

**Table 1:** Baseline characteristics stratified with RI at 1 month and 3 months after transplantation

	Overall	RI < 0.70 1 month	RI < 0.70 1 month	RI ≥ 0.70 1 month	RI ≥ 0.70 1 month	p
		RI < 0.70 3 months	RI ≥ 0.70 3 months	RI < 0.70 3 months	RI ≥ 0.70 3 months	
**Total patients**	1685	661	160	216	648	
**Donor characteristics**						
Cardiovascular death (%)	924 (61.4)	296 (52.4)	103 (70.5)	110 (58.8)	415 (68.4)	< 0.001
Deceased donor (%)	1590 (94.4)	606 (91.7)	150 (93.8)	203 (94.0)	631 (97.4)	< 0.001
Donor age (years)	50.95 (17.54)	42.12 (15.65)	51.86 (16.09)	50.99 (15.36)	59.72 (15.87)	< 0.001
Donor with diabetes (%)	95 (5.7)	14 (2.1)	3 (1.9)	13 (6.1)	65 (10.1)	< 0.001
Donor gender (% Male)	1002 (59.5)	407 (61.6)	90 (56.2)	129 (59.7)	376 (58.0)	0.481
Cold Ischemia (hours)	17.81 (7.95)	17.36 (8.22)	17.41 (7.49)	17.59 (8.23)	18.43 (7.66)	0.085
**Recipient characteristics at time of transplantation**						
Diabetes (%)	263 (15.9)	19 (2.9)	18 (11.2)	31 (14.9)	195 (30.5)	< 0.001
NODAT (%)	214 (12.9)	76 (11.7)	24 (15.1)	23 (11.1)	91 (14.2)	0.379
Hemodialysis time (years)	2.95 (3.34)	3.00 (3.83)	2.79 (2.95)	2.99 (3.33)	2.91 (2.90)	0.902
Age (years)	51.15 (14.78)	41.33 (12.69)	51.00 (12.45)	50.95 (12.88)	61.28 (10.47)	< 0.001
Year of transplantation (%)						< 0.001
1985–1989	44 (2.6)	23 (3.5)	3 (1.9)	6 (2.8)	12 (1.9)	
1990–1999	270 (16.0)	120 (18.2)	28 (17.5)	39 (18.1)	83 (12.8)	
2000–2009	584 (34.7)	257 (38.9)	69 (43.1)	70 (32.4)	188 (29.0)	
2010–2017	787 (46.7)	261 (39.5)	60 (37.5)	101 (46.8)	365 (56.3)	
Gender (% Male)	1074 (63.7)	440 (66.6)	99 (61.9)	149 (69.0)	386 (59.6)	0.019
BMI (kg/m2)	25.31 (4.88)	24.14 (4.59)	24.86 (4.95)	25.18 (4.67)	26.67 (4.91)	< 0.001
Graft rank (%)						0.772
1	1433 (85.0)	551 (83.4)	139 (86.9)	187 (86.6)	556 (85.8)	
2	213 (12.6)	95 (14.4)	19 (11.9)	22 (10.2)	77 (11.9)	
3	37 (2.2)	14 (2.1)	2 (1.2)	7 (3.2)	14 (2.2)	
4	2 (0.1)	1 (0.2)	0 (0.0)	0 (0.0)	1 (0.2)	
Perfusion machine (%)	242 (14.4)	38 (5.7)	12 (7.5)	23 (10.6)	169 (26.1)	< 0.001
Double transplantation (%)	26 (1.5)	2 (0.3)	3 (1.9)	3 (1.4)	18 (2.8)	0.004
DGF (%)	320 (19.0)	88 (13.3)	26 (16.2)	45 (20.8)	161 (24.8)	< 0.001
Thymoglobulin (%)	915 (54.4)	373 (56.6)	89 (55.6)	115 (53.2)	338 (52.2)	0.422
IL2-R antibodies (%)	744 (44.3)	271 (41.2)	68 (42.8)	99 (45.8)	306 (47.4)	0.144
**Recipients characteristics at 3 months**						
SBP (mmHg)	138.54 (15.90)	135.58 (14.86)	135.69 (15.79)	136.80 (14.52)	143.10 (16.45)	< 0.001
DBP (mmHg)	78.79 (10.57)	81.61 (10.03)	78.81 (9.24)	79.96 (9.35)	75.31 (10.88)	< 0.001
PP (mmHg)	59.75 (15.19)	53.96 (12.32)	56.88 (13.79)	56.84 (11.93)	67.80 (15.90)	< 0.001
eGFR (ml/min/1.73 m2)	51.39 (19.09)	56.89 (21.13)	49.71 (16.66)	51.10 (16.56)	45.94 (16.36)	< 0.001
Proteinuria (g/day)	0.80 (8.39)	1.23 (13.41)	0.43 (0.41)	0.55 (0.87)	0.55 (0.70)	0.648
Tacrolimus (%)	823 (55.9)	315 (53.0)	68 (47.6)	109 (57.1)	331 (60.7)	0.010
Ciclosporine (%)	586 (39.8)	267 (44.9)	66 (46.2)	74 (38.7)	179 (32.8)	< 0.001
Steroids (%)	1408 (95.7)	571 (96.1)	135 (95.1)	180 (94.2)	522 (95.8)	0.711
MMF (%)	1193 (81.0)	480 (80.8)	116 (81.1)	149 (78.0)	448 (82.2)	0.651
Azathioprine (%)	234 (15.9)	99 (16.7)	23 (16.1)	34 (17.9)	78 (14.3)	0.602
m-TOR inhibitors (%)	97 (6.6)	19 (3.2)	13 (9.1)	12 (6.2)	53 (9.7)	< 0.001
Resistive index M1	0.70 (0.08)	0.63 (0.05)	0.65 (0.04)	0.73 (0.03)	0.77 (0.06)	< 0.001
Resistive index M3	0.69 (0.08)	0.62 (0.05)	0.73 (0.03)	0.65 (0.04)	0.77 (0.05)	< 0.001
Resistive index M1 > 0.70	864 (51.3)	0 (0.0)	0 (0.0)	216 (100.0)	648 (100.0)	< 0.001
Resistive index M3 > 0.70	808 (48.0)	0 (0.0)	160 (100.0)	0 (0.0)	648 (100.0)	< 0.001

Values are mean (SD) or absolute (percentage) of patients

*NODAT* New Onset Diabetes After transplantation, *DGF* Delayed Graft Function, *BMI* Body Mass Index, *SBP* Systolic Blood Pressure, *DBP* Diastolic Blood Pressure, *PP* Pulse Pressure, *eGFR* estimated Glomerular filtration Rate using MDRD formula, *m-TOR* Mammalian target of rapamycin, *IL2-R* interleukin 2 receptor, *MMF* mycophenolate mofetil

### RI at 1 and 3 months and the risk of death with functioning graft in the whole population and in patients with diabetes mellitus

**Fig. 2:**
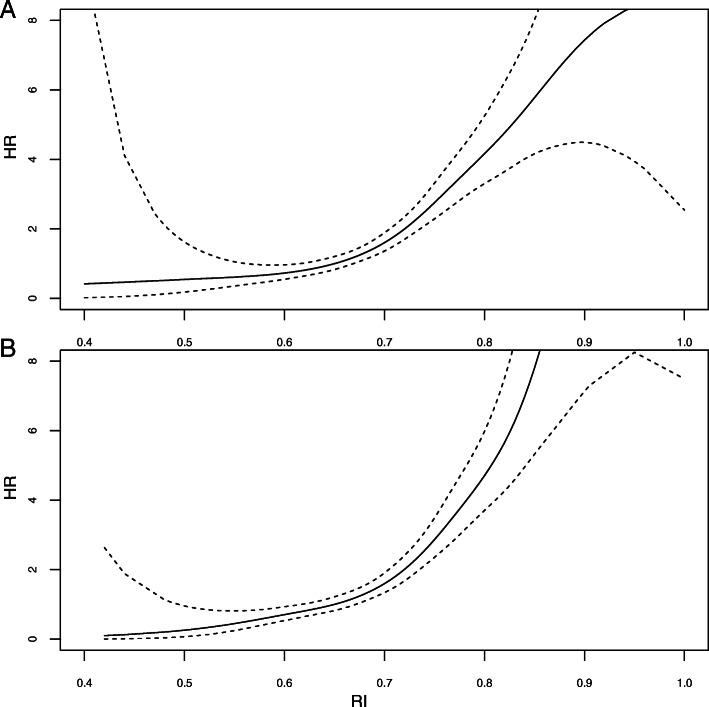
Risk of death with functioning graft according to RI at 1 month (**a**), and according to RI at 3 months (**b**), after kidney transplantation (univariate analysis)

**Fig. 3:**
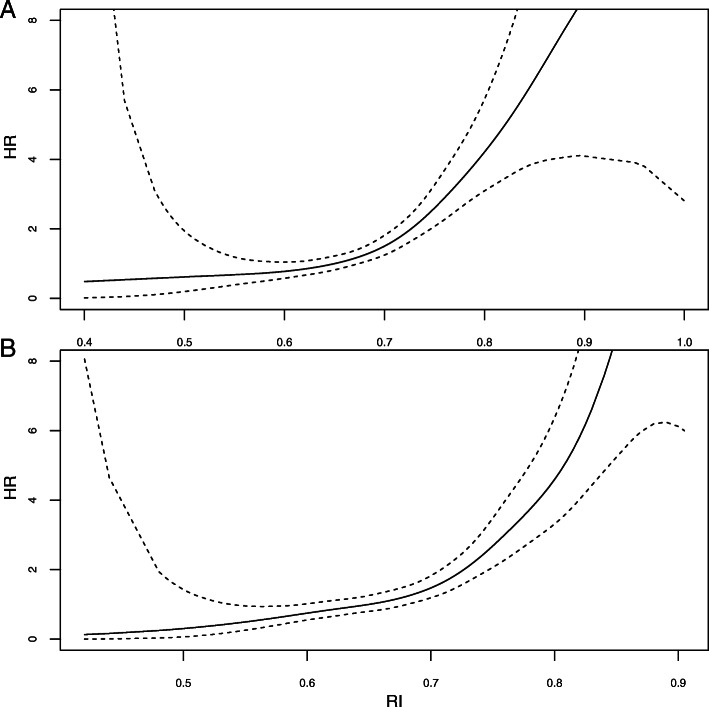
Risk of death with functioning graft in nondiabetic patients according to RI at 1 month (**a**), and RI at 3 months (**b**), after kidney transplantation (univariate analysis)

In the whole population, RI (used as categorical parameter) at 1 month (hazard ratio (HR) = 1.93 [95% confidence interval (95%CI) = 1.65–2.24], *p* < 0.001) and at 3 months (HR = 2.27 [1.91–2.69], p < 0.001) were both associated with an increased risk of DWFG (Table 2). When RI was used as a continuous parameter, we observed that the risk of death increased with increasing RI value both at 1 month (Fig. 2a) and 3 months (Fig. 2b) in the whole population and in nondiabetic patients at 1 month (Fig. 3a) and at 3 months (Fig. 3b).

**Table 2:** Determinants of death with functioning graft in univariate analyses

	HR	p
RI categories (ref = RI < 0.70 both at 1 month & 3 months)	1	
RI < 0.70 at 1 month & RI ≥ 0.70 at 3 months	2.15 [1.29–3.60]	0.003
RI ≥ 0.70 at 1 month & RI < 0.70 at 3 months	1.90 [1.20–3.03]	0.006
RI ≥ 0.70 at 1 month & RI ≥ 0.70 at 3 months	3.77 [2.71–5.24]	< 0.001
**Donor characteristics**		
Cardiovascular death	1.59 [1.16–2.16]	0.003
Donor with diabetes	1.07 [0.54–2.11]	0.850
Donor gender (Male)	1.01 [0.77–1.33]	0.950
Donor Age (per 10 year increase)	1.30 [1.20–1.42]	< 0.001
Donor Age > 60	1.85 [1.44–2.39]	< 0.001
Cold ischemia (per 1 h increase)	1.01 [0.99–1.02]	0.47
**Recipents characteristics at time of transplantation**		
Diabetes	3.44 [2.52–4.70]	< 0.001
NODAT	0.75 [0.66–1.35]	0.75
Hemodialysis time > 1 year	1.38 [0.98–1.92]	0.059
Hemodialysis time (per 1 year increase)	1.02 [0.99–1.06]	0.099
Age (per 10 year increase)	1.92 [1.71–2.16]	< 0.001
Age > 60 years	3.70 [2.85–4.80]	< 0.001
Male gender	1.25 [0.95–1.65]	0.110
BMI > 25	1.68 [1.28–2.20]	< 0.001
BMI (per 5 pt. increase)	1.35 [1.18–1.54]	< 0.001
Year of transplantation (ref = 1985–1989)		
1990–1999	0.98 [0.59–1.64]	0.940
2000–2009	1.03 [0.64–1.66]	0.900
2010–2017	1.42 [0.85–2.40]	0.180
Double transplantation	2.86 [1.11–7.40]	0.030
Perfusion machine	2.49 [1.50–4.15]	< 0.001
DGF	1.38 [1.01–1.89]	0.041
**Recipients characteristics at 3 months**		
SBP > 140 mmHg	1.67 [1.27–2.18]	< 0.001
SBP (per 10 mmHg increase)	1.15 [1.07–1.23]	< 0.001
DBP > 90 mmHg	0.47 [0.24–0.91]	0.026
PB (per 10 mmHg increase)	0.87 [0.77–0.98]	0.024
PP > 50 mmHg	2.12 [1.54–2.91]	< 0.001
PP (per 10 mmHg increase)	1.25 [1.25–1.35]	< 0.001
eGFR < 45 ml/min	1.39 [1.06–1.82]	0.016
eGFR MDRD (per 10 ml/min/1.73 m2 increase)	0.89 [0.82–0.96]	0.024
Tacrolimus	1.18 [0.89–1.57]	0.26
IL2-R antibodies	1.18 [0.89–1.57]	0.250
**Resistive index**		
RIM1 (per 0.1 increase)	1.93 [1.65–2.24]	< 0.001
RIM3 (per 0.1 increase)	2.27 [1.91–2.69]	< 0.001

Values are mean (SD) or absolute (percentage) of patients

*NODAT* New Onset Diabetes After transplantation, *DGF* Delayed Graft Function, *BMI* Body Mass Index, *SBP* Systolic Blood Pressure, *DBP* Diastolic Blood Pressure, *PP* Pulse Pressure, *eGFR* estimated Glomerular filtration Rate using MDRD formula

**Fig. 4:**
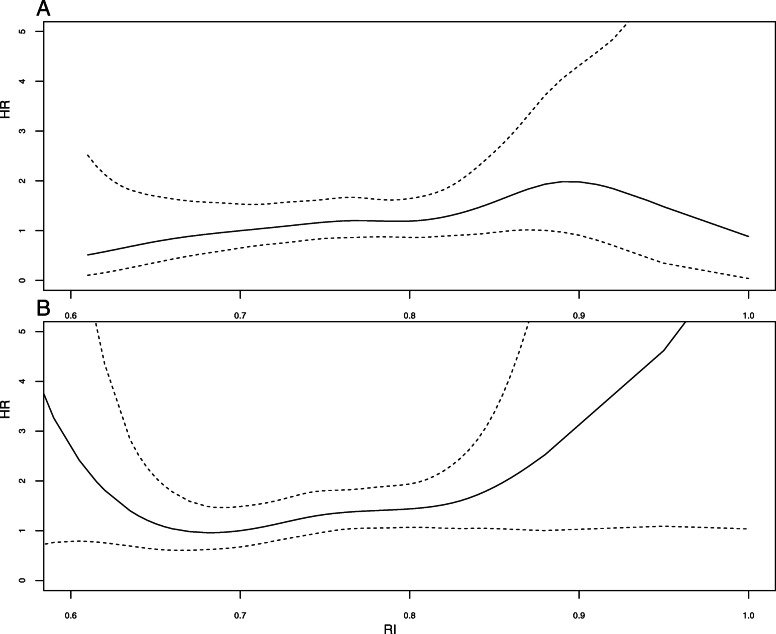
Risk of death with functioning graft in patients with pre-transplant diabetes according to RI at 1 month (**a**), and RI at 3 months (**b**), after kidney transplantation (univariate analysis)

Pre-transplant diabetes was associated with an increased risk of DWFG (HR = 3.44 [2.52–4.70], p < 0.001) (Table 2). RI (used as a continuous variable) measured at 1 (Fig. 4a) and 3 months (Fig. 4b) was not associated with the risk of DFWG in patients with pretransplant diabetes.

### Changes in RI value from 1 to 3 months and risk of death with a functioning graft in the whole population and in patients with pretransplant diabetes

#### Whole population

Individual changes in RI occurred between 1 and 3 months despite the fact that the mean RI value was almost identical at 1 month and 3 months after transplantation: among patients with RI < 0.70 at 1 month, 160 (19.5%) had a RI ≥ 0.70 at 3 months, and among patients with RI ≥ 0.70 at 1 month, 216 (25%) had a RI < 0.70 at 3 months (Table 1).

**Fig. 5:**
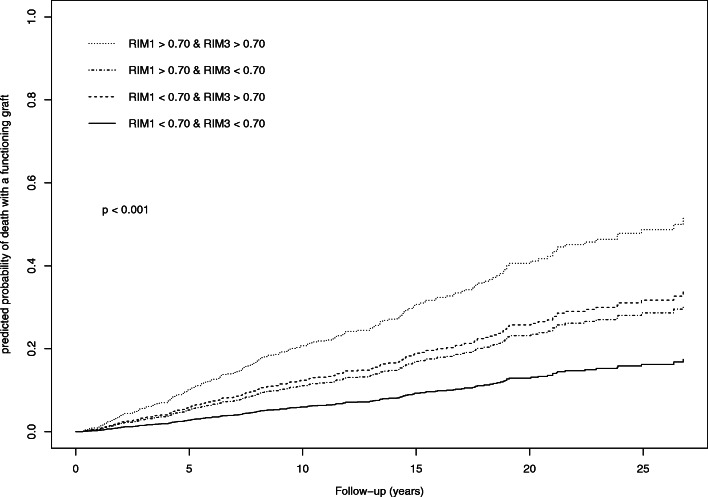
Cumulative incidence of death with functioning graft according to RI variations between 1 month and 3 months after transplantation

Overall, the best survival was observed in the group of patients with RI < 0.70 both at 1 month and 3 months, and the worst survival was found in patients with RI ≥ 0.70, both at 1 and 3 months (HR = 3.77 [2.71–5.24], *p* < 0.001). The risk of DWFG was intermediate for patients with RI < 0.70 at 1 month and RI ≥ 0.70 at 3 months (HR = 2.15 [1.29–3.60], *p* = 0.003) and in those with RI ≥ 0.70 at 1 month and RI < 0.70 at 3 months (HR = 1.90 [1.20–3.03], *p* = 0.006) (Table 2) (Fig. 5).

Consequently, based on the RI value at 1 month, 864/1685 (51.3%) patients would have been considered as “high risk” patients for the risk of DWFG (i.e. RI ≥ 0.70); however, 216 (25.0%) of these 864 “high risk” patients were reclassified as “intermediate risk” patients using RI value both at 1 month and 3 months. Similarly, based on the RI value at 1 month, 821/1685 (48.7%) patients would have been considered as “low risk” patients (i.e. RI < 0.70); however, 160 (19.5%) of these 821 “low risk” patients were reclassified as “intermediate risk” patients using both 1 month and 3 months RI values.

We also used multivariate analysis. A correlation of more than 0.7 was found between recipient age and donor age (r = 0.807), and between systolic blood pressure and pulse pressure at 3 months (r = 0.776). Therefore, donor age and systolic blood pressure were removed from the analysis. In multivariate analyses, RI > 0.70 at 1 months and 3 months (HR = 1.72 [1.07–2.79], *p* = 0.026), as well as RI < 0.70 at 1 month and ≥ 0.70 at 3 months (HR = 1.77 [1.02–3.07], *p* = 0.044), but not RI ≥ 0.70 at 1 months and < 0.70 at 3 months (HR = 1.34 [0.76–2.37], *p* = 0.310) remained a predictor of DWFG (Table 3).

**Table 3:** Association between RI at 1 and 3 months and death with functioning graft in multivariate analysis*

	HR	p
Categories RI (ref = RI < 0.70 both at 1 month & 3 months)	1	
RI < 0.70 at 1 month & RI ≥0.70 at 3 months	1.77 [1.02–3.07]	0.044
RI ≥ 0.70 at 1 month & RI < 0.70 at 3 months	1.34 [0.76–2.37]	0.310
RI ≥ 0.70 at 1 month & RI ≥0.70 at 3 months	1.72 [1.07–2.79]	0.026

Values are mean (SD) or absolute (percentage) of patients

*DGF* Delayed Graft Function, *BMI* Body Mass Index, *SBP* Systolic Blood Pressure, *DBP* Diastolic Blood Pressure, *PP* Pulse Pressure, *eGFR* estimated Glomerular filtration Rate using MDRD formula

*Multivariate analysis adjusted on recipient diabetes, age, donor cardiovascular death, body mass index, perfusion machine, double transplantation, pulse pressure, diastolic blood pressure, delayed graft function, and estimated glomerular filtration rate

#### Impact of pretransplant diabetes

The RI value changed between 1 and 3 months, but this change was different in diabetic and nondiabetic patients: among patients with RI < 0.70 at 1 month, more diabetic than nondiabetic RTR had a RI value ≥0.70 at 3 months (48.6% vs 18.1%, p < 0.001); in contrast, among patients with RI ≥ 0.70 at 1 month, RI was < 0.70 at 3 months in less diabetic than nondiabetic patients (13.7% vs 28.5%, p < 0.001).

Among diabetic RTR, RI ≥ 0.70 at 1 month and 3 months, RI < 0.70 at 1 month and ≥ 0.70 at 3 months and RI ≥ 0.70 at 1 month and < 0.70 at 3 months were not associated with an increased risk of DWFG in univariate analysis. In multivariate analysis, only the group of patients with RI < 0.70 at 1 month and ≥ 0.70 at 3 months had an increased risk of DWFG (vs the group of patients with RI < 0.70 both at 1 month and 3 months) (HR = 4.69 [1.07–20.52], *p* = 0.040) (Table 4).

**Table 4:** Association between RI at 1 and 3 months and death with functioning graft in patients with pre-transplant diabetes

	Univariate		Multivariate^a^	
	HR	p	HR	p
Catégories RI (ref = RI < 0.70 both at 1 month & 3 months)	1		1	
RI < 0.70 at 1 month & RI ≥ 0.70 at 3 months	3.67 [0.94–14.32]	0.061	4.69 [1.07–20.52]	0.040
RI ≥ 0.70 at 1 month & RI < 0.70 at 3 months	2.16 [0.63–7.41]	0.220	1.63 [0.42–6.30]	0.480
RI ≥ 0.70 at 1 month & RI ≥ 0.70 at 3 months	2.15 [0.74–6.25]	0.160	1.34 [0.43–4.20]	0.610

Values are mean (SD) or absolute (percentage) of patients

^a^Multivariate analysis adjusted on age, donor cardiovascular death, body mass index, perfusion machine, double transplantation, pulse pressure, diastolic blood pressure, delayed graft function, and estimated glomerular filtration rate

### Sensitivity analysis

#### Changes in RI value from 1 to 3 months and risk of DWFG graft using a threshold of 0.75

1217 patients (72.2%) at 1 month, and 468 patients (27.7%) at 3 months had a RI < 0.75 (Table 1). Among patients with RI < 0.75 at 1 month, 140 (11.5%) had a RI ≥ 0.75 at 3 months, and among patients with RI ≥ 0.75 at 1 month, 164 (35.0%) had a RI < 0.75 at 3 months (Supplementary Table 1).

Best survival was also observed in patients with RI < 0.75 at 1 month and 3 months after transplantation. RI > 0.75 at 1 months and 3 months remained a predictor of DWFG (HR = 3.77 [2.73–5.21], p < 0.001), as well as RI < 0.75 at 1 month and ≥ 0.75 at 3 months (HR = 3.48 [2.33–5.18], p < 0.001), and RI ≥ 0.75 at 1 month and < 0.75 at 3 months (HR = 2.53, [1.73–3.68], p < 0.001).

In multivariate analyses: the risk of DWFG was associated with RI ≥ 0.75 at 1 months and 3 months (HR = 1.72 [1.06–2.78], *p* = 0.027) and increasing RI from 1 month to 3 months (HR = 2.30 [1.41–2.74], p < 0.001) but not decreasing RI from 1 month to 3 months (HR = 1.45 [0.88–2.40], *p* = 0.140) (Supplementary Table 2).

Based on the RI value at 1 month, 468/1685 (27.8%) patients would have been considered as “high risk” patients for the risk of death with functioning graft (i.e. RI ≥ 0.75); however, 164 (35%) of these 468 “high risk” patients were reclassified as “intermediate risk” patients using RI values at 1 and 3 months. Similarly, based on the RI value at 1 month, 1217/1685 (72.2%) patients would have been considered as “low risk” patients (i.e. RI < 0.75); however, 140 (11.5%) of these 1217 “low risk” patients were reclassified as “intermediate risk” patients using RI values at 1 and 3 months.

## Discussion

In the present study, we confirmed that RI as a continuous variable was correlated to DWFG, whether it is measured at 1 or 3 months after kidney transplantation. Then we showed that the short-term change in RI between 1 month and 3 months after transplantation was also associated with death with functioning graft. In diabetic patients the results were quite different. First, the relationship between RI at 1 month or at 3 months and the risk of DWFG was not the same in diabetic and in nondiabetic patients. Second, significantly more diabetic than nondiabetic patients with RI > 0.70 at 1 month remained with RI > 0.70 at 3 months whereas significantly less diabetic than nondiabetic patients with RI < 0.70 at 1 month remained with a RI < 0.70 at 3 months. Then, among diabetic patients, an increased risk of DWFG was observed only in those with low RI at 1 month and high RI at 3 months. We found that the variation of RI could refine its prognostic value. Indeed, in all patients, compared to low RI at 1 month and 3 months, high RI at 1 month and 3 months was always associated with a higher risk of DWFG. Increasing RI (meaning low RI at 1 month and high RI at 3 months) was also always associated with a higher risk of DWFG. On the other hand, depending on the cut-off, decreasing RI (meaning high RI at 1 month and low RI at 3 months) could be of better prognosis, as it was not always associated with a higher risk of DWFG. Moreover, in patients with pre-transplant diabetes, only increasing RI was associated with a higher risk of DWFG in multivariate analyses.

High RI is observed in patients with DGF, in acute rejection, and also in all causes of acute tubular necrosis [17]. On the other hand, many studies suggest that RI is related to systemic vascular alterations, and poorly associated with renal vascular resistance [18–21]. Studies showed that it was increased in patients with atherosclerosis, and with diabetic nephropathy [22, 23]. Diabetic patients suffer the vascular consequences of chronic glucotoxicity [24]. These complications imply both systemic and renal vascularisation; hence, the impact on RI. In a previous study, we showed that RI does not have the same prognostic value in diabetic patients receiving a kidney transplant [4]. Indeed, in the present study, we found a very different association between RI as a continuous variable and the risk of DWFG, which confirms that RI is more difficult to interpret in patients with pre-transplant diabetes.

In our previous studies [4, 20], we only analysed RI measured at 3 months after kidney transplantation. The prognostic value of resistance index after kidney transplantation is well-known, but authors diverge on the best timing of the RI measurement [5, 6, 25].

Some authors also made the hypothesis that the variation of RI would be of interest [26]. We also found that RI at 3 months was not a good predictor of DWFG in patients with pre-transplant diabetes. We hypothesized that the increase of RI was less important in diabetic patients because RI was higher in diabetic patients than in non-diabetic patients, due to aortic stiffness, and that it could explain the absence of prognostic value of RI in patients with pre-transplant diabetes. However, in the present study, in patients with pre-transplant diabetes, increasing RI was associated with DWFG, which means that patients with a low RI at 1 month and high RI at 3 months had a worst prognosis than others. In this way, the evolution of RI between 1 month and 3 months refines its prognostic value.

High RI is supposedly correlated to kidney recipient arterial stiffness, hence its long-term prognostic value. We hypothesize that conversely, RI changes between 1 and 3 months could be the result of local acute changes in the graft, RI at 1 month being more linked to the graft and less to the recipient than RI at 3 months. Initial high RI could be due to acute complications of the graft, like DGF or NTA, and low RI at 3 months could reflect the vascular environment of the donor.

Our study represents one of the largest cohorts of renal transplant recipients focused on RI variations early after transplantation. Regarding Doppler indices, there is a good expertise on measuring and studying this parameter, as these parameters are studied in our hospital since the early seventies [9].

It also has limitations. It is a retrospective monocentric study therefore our findings would need replication. We could not differentiate cardiovascular and non-cardiovascular death: the difference in the prognostic value of RI may be different for cardiovascular death. Also, we missed data concerning cardiovascular history of patients, and history of medications known to reduce cardiovascular risk (antihypertensive treatments, aspirin, statins) [27]. As these data were only available from 2016, and therefore in patients older, with a higher frequency of diabetes, and more extended criteria donors, the related bias seemed too important. We also were not able to provide data regarding diabetes severity. Finally, it was not possible to provide the inter-observer variability of the RI measure. However, some studies showed a good reproducibility of RI measurements [28, 29]. It was also not possible to provide the inter-device variability of RI measurements, but there were no specific differences notified in the accuracy of the devices’ measurements.

## Conclusions

In conclusion, our study indicates that high RI at different time early after transplantation is a strong predictor of DWFG graft in renal transplant patients, but has a different interpretation in diabetic patients. Its variation between 1 month and 3 months also refines its prognostic value. These findings could be interesting in the management of patients early after transplantation. Non-diabetic patients with high RI at 1 month, 3 months, or both, as well as diabetic patients with increasing RI between 1 month and 3 months should benefit from improved cardiovascular prevention and follow-up.

## Supplementary Information

**Additional file 1: Supplementary Table 1** Baseline characteristics stratified with RI at 1 month and 3 months after transplantation using a threshold of 0.75.

**Additional file 2: Supplementary Table 2**. Determinants of death with a functioning graft in multivariate analysis using a threshold of 0.75.

**Additional file 3: Supplementary Table 3**. RI according to diabetes status.

## Data Availability

The datasets used and/or analysed during the current study are available from the corresponding author on reasonable request.

## Abbreviations

ANOVA: Analysis of variance
BMI: Body mass index
CI: Confidence interval
CICR: Cumulative incidence competing risk
DBP: Diastolic blood pressure
DSA: Donor specific antigen
DWFG: Death with a functioning graft
eGFR: Estimated glomerular filtration rate
ESRD: End-stage renal disease
HR: Hazard ratio
MMF: Mycophenolate mofetyl
m-TOR: Mammalian-target of rapamycin
NODAT: New onset diabetes after transplantation
PP: Pulse pressure
RI: Renal resistive index
SBP: Systolic blood pressure
RTR: Renal transplant recipients
