# Supplementary material for: Early changes in renal resistive index and mortality in diabetic and nondiabetic kidney transplant recipients: a cohort study
Source: BMC Nephrol. 2021 Feb 19;22:62. doi: 10.1186/s12882-021-02263-8 (PMC7893742; doi:10.1186/s12882-021-02263-8)
Supplement: Supplementary file 1 — Additional file 1: Supplementary Table 1 Baseline characteristics stratified with RI at 1 month and 3 months after transplantation using a threshold of 0.75. [file 12882_2021_2263_MOESM1_ESM.docx]

Early changes in renal resistive index and mortality in diabetic and nondiabetic kidney transplant recipients : a cohort study

Changes in resistive index and mortality

Jean-Baptiste DE FREMINVILLE^1,6^, Louis-Marie VERNIER^4^, Jérome ROUMY^2,5^, Frédéric PATAT^2,5,6^, Philippe GATAULT^1,3,6^, Bénédicte SAUTENET^1,6^, Christelle BARBET^1^, Hélène LONGUET^1^, Elodie MERIEAU^1^, Matthias BUCHLER^1,3,6^, Jean-Michel HALIMI^1,3,6^.

1 - Néphrologie-Immunologie Clinique, Hôpital Bretonneau, CHU Tours, Tours, France

2 - Imagerie Médicale, Hôpital Bretonneau, CHU Tours, Tours, France

3 - EA4245, University of Tours, Tours, France

4 - Néphrologie-Dialyse, Centre de santé pluridisciplinaire, Le Mans, France

5 - CIC-IT 1415, CHU Tours

6 – University of Tours, Tours, France

**Corresponding author :**

de Freminville Jean-Baptiste.

E-mail: [jean.de-freminville@polytechnique.org](mailto:jean.de-freminville@polytechnique.org)
ORCID : <https://orcid.org/0000-0003-3829-9506>

Twitter : @jbdefrem

Supplementary table 1. Baseline characteristics stratified with RI at 1 month and 3 months after transplantation using a threshold of 0.75

|  | **Overall** | **RI < 0.75 1 month** | **RI < 0.75 1 month** | **RI** ≥**0.75 1 month** | **RI** ≥**0.75 1 month** | **p** |
| --- | --- | --- | --- | --- | --- | --- |
|  |  | **RI < 0.75 3 months** | **RI** ≥**0.75 3 months** | **RI < 0.75 3 months** | **RI** ≥**0.75 3 months** |  |
| **Total patients** | 1685 | 1077 | 140 | 164 | 304 |  |
| **Donor characteristics** |  |  |  |  |  |  |
| Cardiovascular death (%) | 924 (61.4) | 535 (57.5) | 88 (66.7) | 89 (59.7) | 212 (72.4) | <0.001 |
| Deceased donor (%) | 1590 (94.4) | 995 (92.4) | 135 (96.4) | 161 (98.2) | 299 (98.4) | <0.001 |
| Donor age (years) | 50.95 (17.54) | 45.97 (16.27) | 58.29 (15.50) | 53.87 (16.76) | 63.63 (15.12) | <0.001 |
| Donor with diabetes (%) | 95 (5.7) | 32 (3.0) | 11 (7.9) | 14 (8.6) | 38 (12.5) | <0.001 |
| Donor gender (% Male) | 1002 (59.5) | 649 (60.3) | 83 (59.3) | 94 (57.3) | 176 (57.9) | 0.823 |
| Cold Ischemia (hours) | 17.81 (7.95) | 17.51 (8.28) | 19.09 (8.01) | 19.01 (7.67) | 17.64 (6.70) | 0.030 |
| **Recipient characteristics at time of transplantation** | | |  |  |  |  |
| Diabetes (%) | 263 (15.9) | 64 (6.0) | 33 (23.9) | 41 (26.3) | 125 (41.4) | <0.001 |
| NODAT (%) | 214 (12.9) | 127 (12.0) | 24 (17.4) | 21 (13.3) | 42 (13.9) | 0.321 |
| Hemodialysis time (years) | 2.95 (3.34) | 2.90 (3.43) | 2.79 (3.12) | 3.49 (3.89) | 2.90 (2.75) | 0.211 |
| Age (years) | 51.15 (14.78) | 45.36 (13.45) | 59.71 (11.12) | 56.37 (12.89) | 64.92 (8.19) | <0.001 |
| Year of transplantation (%) |  |  |  |  |  | <0.001 |
| 1985-1989 | 44 (2.6) | 31 (2.9) | 3 (2.1) | 8 (4.9) | 2 (0.7) |  |
| 1990-1999 | 270 (16.0) | 196 (18.2) | 12 (8.6) | 23 (14.0) | 39 (12.8) |  |
| 2000-2009 | 584 (34.7) | 410 (38.1) | 58 (41.4) | 52 (31.7) | 64 (21.1) |  |
| 2010-2017 | 787 (46.7) | 440 (40.9) | 67 (47.9) | 81 (49.4) | 199 (65.5) |  |
| Gender (% Male) | 1074 (63.7) | 706 (65.6) | 80 (57.1) | 100 (61.0) | 188 (61.8) | 0.159 |
| BMI (kg/m2) | 25.31 (4.88) | 24.54 (4.59) | 26.24 (5.87) | 26.17 (4.82) | 27.15 (4.82) | <0.001 |
| Graft rank (%) |  |  |  |  |  | 0.863 |
| 1 | 1433 (85.0) | 913 (84.8) | 117 (83.6) | 140 (85.4) | 263 (86.5) |  |
| 2 | 213 (12.6) | 135 (12.5) | 22 (15.7) | 21 (12.8) | 35 (11.5) |  |
| 3 | 37 (2.2) | 27 (2.5) | 1 (0.7) | 3 (1.8) | 6 (2.0) |  |
| 4 | 2 (0.1) | 2 (0.2) | 0 (0.0) | 0 (0.0) | 0 (0.0) |  |
| Perfusion machine (%) | 242 (14.4) | 86 (8.0) | 31 (22.1) | 24 (14.6) | 101 (33.2) | <0.001 |
| Double transplantation (%) | 26 (1.5) | 9 (0.8) | 2 (1.4) | 4 (2.5) | 11 (3.6) | 0.005 |
| DGF (%) | 320 (19.0) | 136.23 (14.92) | 138.07 (18.06) | 141.64 (13.59) | 145.91 (17.08) | <0.001 |
| Thymoglobulin (%) | 915 (54.4) | 80.85 (9.85) | 76.74 (11.00) | 77.19 (10.23) | 72.76 (10.60) | <0.001 |
| IL2-R antibodies (%) | 744 (44.3) | 55.38 (12.60) | 61.33 (16.16) | 64.45 (13.99) | 73.15 (15.73) | <0.001 |
| **Recipients characteristics at 3 months** | |  |  |  |  |  |
| SBP (mmHg) | 138.54 (15.90) | 157 (14.6) | 30 (21.4) | 44 (26.8) | 89 (29.3) | <0.001 |
| DBP (mmHg) | 78.79 (10.57) | 54.47 (19.68) | 46.67 (17.74) | 47.72 (17.17) | 43.91 (15.25) | <0.001 |
| PP (mmHg) | 59.75 (15.19) | 0.94 (10.58) | 0.53 (0.58) | 0.64 (1.21) | 0.57 (0.56) | 0.930 |
| eGFR (ml/min/1.73 m2) | 51.39 (19.09) | 509 (53.1) | 72 (57.6) | 80 (58.0) | 162 (64.5) | 0.011 |
| Proteinuria (g/day) | 0.80 (8.39) | 423 (44.1) | 41 (32.8) | 49 (35.5) | 73 (29.1) | <0.001 |
| Tacrolimus (%) | 823 (55.9) | 917 (95.7) | 117 (93.6) | 132 (95.7) | 242 (96.4) | 0.653 |
| Ciclosporine (%) | 586 (39.8) | 768 (80.1) | 112 (89.6) | 107 (77.5) | 206 (82.1) | 0.051 |
| Steroids (%) | 1408 (95.7) | 165 (17.2) | 10 (8.0) | 27 (19.6) | 32 (12.7) | 0.016 |
| MMF (%) | 1193 (81.0) | 45 (4.7) | 14 (11.2) | 12 (8.7) | 26 (10.4) | 0.001 |
| Azathioprine (%) | 234 (15.9) | 601 (55.9) | 73 (52.1) | 89 (54.3) | 152 (50.0) | 0.303 |
| m-TOR inhibitors (%) | 97 (6.6) | 455 (42.4) | 66 (47.5) | 74 (45.4) | 149 (49.0) | 0.176 |
| Resistive index M1 | 0.70 (0.08) | 0.65 (0.06) | 0.71 (0.03) | 0.78 (0.04) | 0.81 (0.05) | <0.001 |
| Resistive index M3 | 0.69 (0.08) | 0.65 (0.06) | 0.78 (0.03) | 0.70 (0.04) | 0.80 (0.04) | <0.001 |
| Resistive index M1 > 0.75 | 864 (51.3) | 0 (0.0) | 140 (100.0) | 0 (0.0) | 304 (100.0) | <0.001 |
| Resistive index M3 > 0.75 | 808 (48.0) | 0 (0.0) | 0 (0.0) | 164 (100.0) | 304 (100.0) | <0.001 |

Values are mean (SD) or absolute (percentage) of patients

NODAT : New Onset Diabetes After transplantation; DGF : Delayed Graft Function ; BMI : Body Mass Index ; SBP : Systolic Blood Pressure ; DBP : Diastolic Blood Pressure ; PP : Pulse Pressure ; eGFR : estimated Glomerular filtration Rate using MDRD formula; m-TOR : Mammalian target of rapamycin ; IL2-R : interleukin 2 receptor ; MMF : mycophenolate mofetil
