# Supplementary material for: Early changes in renal resistive index and mortality in diabetic and nondiabetic kidney transplant recipients: a cohort study
Source: BMC Nephrol. 2021 Feb 19;22:62. doi: 10.1186/s12882-021-02263-8 (PMC7893742; doi:10.1186/s12882-021-02263-8)
Supplement: Supplementary file 2 — Additional file 2: Supplementary Table 2. Determinants of death with a functioning graft in multivariate analysis using a threshold of 0.75. [file 12882_2021_2263_MOESM2_ESM.docx]

Early changes in renal resistive index and mortality in diabetic and nondiabetic kidney transplant recipients : a cohort study

Changes in resistive index and mortality

Jean-Baptiste DE FREMINVILLE^1,6^, Louis-Marie VERNIER^4^, Jérome ROUMY^2,5^, Frédéric PATAT^2,5,6^, Philippe GATAULT^1,3,6^, Bénédicte SAUTENET^1,6^, Christelle BARBET^1^, Hélène LONGUET^1^, Elodie MERIEAU^1^, Matthias BUCHLER^1,3,6^, Jean-Michel HALIMI^1,3,6^.

1 - Néphrologie-Immunologie Clinique, Hôpital Bretonneau, CHU Tours, Tours, France

2 - Imagerie Médicale, Hôpital Bretonneau, CHU Tours, Tours, France

3 - EA4245, University of Tours, Tours, France

4 - Néphrologie-Dialyse, Centre de santé pluridisciplinaire, Le Mans, France

5 - CIC-IT 1415, CHU Tours

6 – University of Tours, Tours, France

**Corresponding author email:**

de Freminville Jean-Baptiste. E-mail: [jean.de-freminville@polytechnique.org](mailto:jean.de-freminville@polytechnique.org)
<https://orcid.org/0000-0003-3829-9506>

Supplementary table 2. Determinants of death with a functioning graft in multivariate analysis using a threshold of 0.75

|  | HR | p |
| --- | --- | --- |
| Catégories RI (ref = RI < 0.75 both at 1 month & 3 months) | 1 |  |
| RI < 0.75 at 1 month & RI ≥ 0.75 at 3 months | 2.30 [1.41-3.74] | <0.001 |
| RI ≥ 0.75 at 1 month & RI < 0.75 at 3 months | 1.45 [0.88-2.40] | 0.140 |
| RI ≥ 0.75 at 1 month & RI ≥ 0.75 at 3 months | 1.72 [1.06-2.78] | 0.027 |

Values are mean (SD) or absolute (percentage) of patients

DGF : Delayed Graft Function ; BMI : Body Mass Index ;; SBP : Systolic Blood Pressure ; DBP : Diastolic Blood Pressure ; PP : Pulse Pressure ; eGFR : estimated Glomerular filtration Rate using MDRD formula

*Multivariate analysis adjusted on diabetes, age, donor cardiovascular death, body mass index, perfusion machine, double transplantation, pulse pressure, diastolic blood pressure, delayed graft function, and estimated glomerular filtration rate
