# Supplementary material for: Early changes in renal resistive index and mortality in diabetic and nondiabetic kidney transplant recipients: a cohort study
Source: BMC Nephrol. 2021 Feb 19;22:62. doi: 10.1186/s12882-021-02263-8 (PMC7893742; doi:10.1186/s12882-021-02263-8)
Supplement: Supplementary file 3 — Additional file 3: Supplementary Table 3. RI according to diabetes status. [file 12882_2021_2263_MOESM3_ESM.docx]

Early changes in renal resistive index and mortality in diabetic and nondiabetic kidney transplant recipients : a cohort study

Changes in resistive index and mortality

Jean-Baptiste DE FREMINVILLE^1,6^, Louis-Marie VERNIER^4^, Jérome ROUMY^2,5^, Frédéric PATAT^2,5,6^, Philippe GATAULT^1,3,6^, Bénédicte SAUTENET^1,6^, Christelle BARBET^1^, Hélène LONGUET^1^, Elodie MERIEAU^1^, Matthias BUCHLER^1,3,6^, Jean-Michel HALIMI^1,3,6^.

1 - Néphrologie-Immunologie Clinique, Hôpital Bretonneau, CHU Tours, Tours, France

2 - Imagerie Médicale, Hôpital Bretonneau, CHU Tours, Tours, France

3 - EA4245, University of Tours, Tours, France

4 - Néphrologie-Dialyse, Centre de santé pluridisciplinaire, Le Mans, France

5 - CIC-IT 1415, CHU Tours

6 – University of Tours, Tours, France

**Corresponding author email:**

de Freminville Jean-Baptiste. E-mail: [jean.de-freminville@polytechnique.org](mailto:jean.de-freminville@polytechnique.org)
<https://orcid.org/0000-0003-3829-9506>

| Supplementary table 3. RI according to diabetes status | | |  |
| --- | --- | --- | --- |
|  |  |  |  |
|  |  | **Overall** | |
|  |  | **Recipient Diabetes -** | **Recipient Diabetes +** |
|  | Resistive index M1 | 0.69 (0.08) | 0.77 (0.07) |
|  | Resistive index M3 | 0.68 (0.08) | 0.76 (0.07) |
|  | Resistive index M1 > 0.70 | 621 (44.6) | 226 (85.9) |
|  | Resistive index M3 > 0.70 | 586 (42.1) | 213 (81.0) |
|  | Resistive index M1 > 0.75 | 282 (20.2) | 158 (60.1) |
|  | Resistive index M3 > 0.75 | 292 (21.0) | 166 (63.1) |
